# The Vermont School Journal

**Published:** 1864-05

**Authors:** 


THE VERMONT SCHOOL JOURNAL. *Devoted to the Educational interests of the State Published under the sanction of the Vermont State Teachers' Association.*—This is a neat, well gotten up and well filled educational journal, one that will be pleasing and interesting to the friends of education everywhere.

It is filled with good articles that are interesting and instructive to all, and especially to teachers. It will afford us much pleasure to receive it regularly.

